# Correlation of tumour subtype with long-term outcome in small breast carcinomas: a Swedish population-based retrospective cohort study

**DOI:** 10.1007/s10549-022-06691-4

**Published:** 2022-08-06

**Authors:** Gunilla Rask, Anoosheh Nazemroaya, Malin Jansson, Charlotta Wadsten, Greger Nilsson, Carl Blomqvist, Lars Holmberg, Fredrik Wärnberg, Malin Sund

**Affiliations:** 1 Department of Medical Biosciences/Pathology, Umeå University, Umeå, Sweden; 2 Department of Surgery and Perioperative Sciences/Surgery, Umeå University, Umeå, Sweden; 3 Department of Immunology, Genetics and Pathology, Section of Experimental and Clinical Oncology, Uppsala University, University Hospital, Uppsala, Sweden; 4 Department of Oncology, Gävle Hospital, Gävle, Sweden; 5 Department of Oncology, Visby Hospital, Visby, Sweden; 6 Department of Oncology, University of Helsinki and Helsinki University Hospital, Helsinki, Finland; 7 Department of Oncology, Örebro University Hospital, Örebro, Sweden; 8 Translational Oncology and Urology Research (TOUR), School of Cancer and Pharmaceutical Sciences, King’s College London, London, UK; 9 Department of Surgical Sciences, Uppsala University, Uppsala, Sweden; 10 Department of Clinical Sciences, Department of Surgery, Sahlgrenska Academy, University of Gothenburg, Gothenburg, Sweden; 11 Department of Surgery, University of Helsinki and Helsinki University Hospital, Helsinki, Finland

**Keywords:** Breast cancer, Molecular subtypes, TMA, Long-term outcome

## Abstract

**Purpose:**

To investigate if molecular subtype is associated with outcome in stage 1 breast cancer (BC).

**Methods:**

Tissue samples from 445 women with node-negative BC ≤ 15 mm, treated in 1986–2004, were classified into surrogate molecular subtypes [Luminal A-like, Luminal B-like (HER2−), HER2-positive, and triple negative breast cancer (TNBC)]. Information on treatment, recurrences, and survival were gathered from medical records.

**Results:**

Tumour subtype was not associated with overall survival (OS). Luminal B-like (HER2−) and TNBC were associated with higher incidence of distant metastasis at 20 years (Hazard ratio (HR) 2.26; 95% CI 1.08–4.75 and HR 3.24; 95% CI 1.17–9.00, respectively). Luminal B-like (HER2−) and TNBC patients also had worse breast cancer-specific survival (BCSS), although not statistically significant (HR 1.53; 95% CI 0.70–3.33 and HR 1.89; 95% CI 0.60–5.93, respectively). HER2-positive BC was not associated with poor outcome despite no patient receiving HER2-targeted therapy, with most of these tumours being ER+.

**Conclusions:**

Stage 1 TNBC or Luminal B-like (HER2−) tumours behave more aggressively. Women with HER2+/ER+ tumours do not have an increased risk of distant metastasis or death, absent targeted treatment.

**Supplementary Information:**

The online version contains supplementary material available at 10.1007/s10549-022-06691-4.

## Introduction

Most women with breast cancer (BC) are diagnosed with stage 1 disease in countries with generally available mammography screening and programmes for early detection [1]. As a result, focus has shifted from clinical stage to tumour biology or molecular subtype of breast cancer when deciding on adjuvant systemic therapy. The surrogate molecular subtypes used in clinical practice are based on those originally described by Sørlie [2] and include Luminal A-like, Luminal B-like (HER2−), HER2-positive (HER2+) and triple negative breast cancer (TNBC). All subtypes besides Luminal A implicate the patient is considered for adjuvant chemotherapy, with addition of targeted anti-HER2-therapy for the HER2+ tumours. However, most studies showing worse outcomes for these tumours and/or benefit of adjuvant chemotherapy and targeted anti-HER2 therapy included women with more advanced clinical stages of BC [3–5]. There are few studies on the potential independent prognostic value of tumour subtypes in women with small lymph node negative BC [6–10]. In these patients, the treatment benefit needs to be put into perspective of treatment induced morbidity since most women are long-term survivors. The prognostic value of different subtypes is also dependent on the length of follow-up, because the natural course of BC varies depending on subtype.

This study investigates the association of surrogate molecular subtypes with survival outcomes and recurrence in a cohort of Swedish women treated for small, lymph node negative BC between 1986–2004, a time period before multimodal treatment protocols were routine and before HER2-targeted therapy was approved for adjuvant BC treatment in Sweden.

## Materials and methods

### Study cohort and generation of tissue microarray (TMA)

The study cohort includes all women identified through the regional breast cancer quality of care registry and operated for unifocal BC with a radiological tumour size ≤ 15 mm at Uppsala university hospital or Västerås hospital between 1986 and 2004. The diagnosis and tumour size were verified through medical records. Additionally, the cohort includes women with breast carcinoma of any size operated at Uppsala university hospital 1986–2004 where the pathology report stated that there was an in situ-component as well as an invasive tumour. To generate a tissue microarray (TMA), we took two 1 mm core biopsies from the formalin fixed, paraffin embedded surgical resection specimens from each patient and embedded them in a recipient tissue block. A fully annotated pseudonymized clinical database included information on baseline characteristics, treatments, relapses, and causes of death with data collected from a review of medical records every other year until March 2015. For the present study, we excluded tumours where the final size on histology was > 15 mm, or there were metastases to axillary lymph nodes. The Regional Ethics Committee of Uppsala approved the study (Record No. 99 422, 2005:118, and 2005:118/2).

### Histology

The TMAs were sectioned and stained for haematoxylin–eosin, oestrogen receptor (ER), progesterone receptor (PR), Ki-67 and HER2 at the Department of Clinical pathology of Umeå University Hospital, using externally validated protocols according to clinical routine. In each run, appropriate external controls were included [tonsil, prostate, endometrium, cervical tissue as well as breast tumor samples with known expression of HER2 (0, 1+, 2+, and 3+)]. For HER2 both immunohistochemistry and silver in situ hybridization (SISH) were performed. Two subspecialized breast pathologists at Umeå University Hospital (a tertiary care centre with approximately 1000 breast cancer cases/year), reviewed the slides. Expression of ER, PR, Ki67, and HER2 were scored separately by either pathologist, but the haematoxylin–eosin slides first separately and then together to reach consensus on the nuclear grade. For ER and PR, tumours were scored as positive (≥ 10%) or negative (< 10%). ER+ tumours with nuclear grade 2 were divided into low (0–13%), intermediate (14–19%) and high (> 20%) Ki67. If a tumour had positive PR but missing an ER value because of technical reasons, the tumour was considered ER positive as well. HER2 IHC was scored as 0–3+ and HER2 SISH as amplified/non-amplified respectively, both according to College of American Pathologists (CAP) guidelines 2018 [11]. Nuclear grade was scored 1–3 according to Elston and Ellis [12]

### Surrogate molecular subtypes

Based on the surrogate classification suggested by St Gallen and revised by Maissonneuve and Ehinger [13, 14] we divided the tumours into four subtypes as defined below. We used nuclear grade instead of histologic grade since neither tubule formation nor mitotic activity can be adequately assessed in TMA cores of this size.

- Luminal A-like (LumA): ER+ or PR+ with nuclear grade 1 *or* nuclear grade 2 with low Ki67 *or* nuclear grade 2 with intermediate Ki67 and PR+
- Luminal B-like (HER2−) (LumB): ER+ or PR+ with nuclear grade 3 *or* nuclear grade 2 with high Ki67 *or* nuclear grade 2 with intermediate Ki67 and PR−
- HER2-positive (HER2+): HER2-staining 3+ by IHC and/or amplified by SISH.
- Triple negative (TNBC): ER−, PR−, and HER2−.

### Statistics

To compare baseline characteristics between the four groups, we used chi-square test or one-way analysis of variance (ANOVA). Primary outcomes were overall survival (OS), breast cancer specific survival (BCSS) and recurrence-free survival (RFS). Secondary outcomes were cumulative incidences of locoregional recurrence and distant metastasis. OS was defined as time from surgery to death from any cause. BCSS was defined as time from surgery to death primarily caused by BC as judged by the researcher reviewing the patients’ medical records. Recurrence was defined as locoregional recurrence or distant metastasis as recorded in the medical records. RFS was defined as time from surgery to recurrence, and patients who died before recurrence were censored at the time of death. The reviewer of the medical records had no information on the subtype classification used in this study. The analyses of RFS and BCSS censored patients at the time of contralateral BC. The analysis of locoregional recurrence censored patients at the time of distant metastasis before locoregional recurrence. The analysis of distant metastasis censored patients at the time of contralateral BC before distant metastasis. To analyse survival outcomes and cumulative incidences, we used Kaplan–Meier curve statistics and compared differences in survival between tumour subtypes with the log rank test. *p*-values ≤ 0.05 were considered statistically significant.

Multivariable analysis with Cox regression for OS, RFS, and BCSS included age, tumour size, and mode of detection. We used SPSS statistics v.26 and STATA IC v 15 for the statistical analyses.

## Results

### Clinical characteristics of the study cohort and surrogate molecular subtypes

**Fig. 1:**
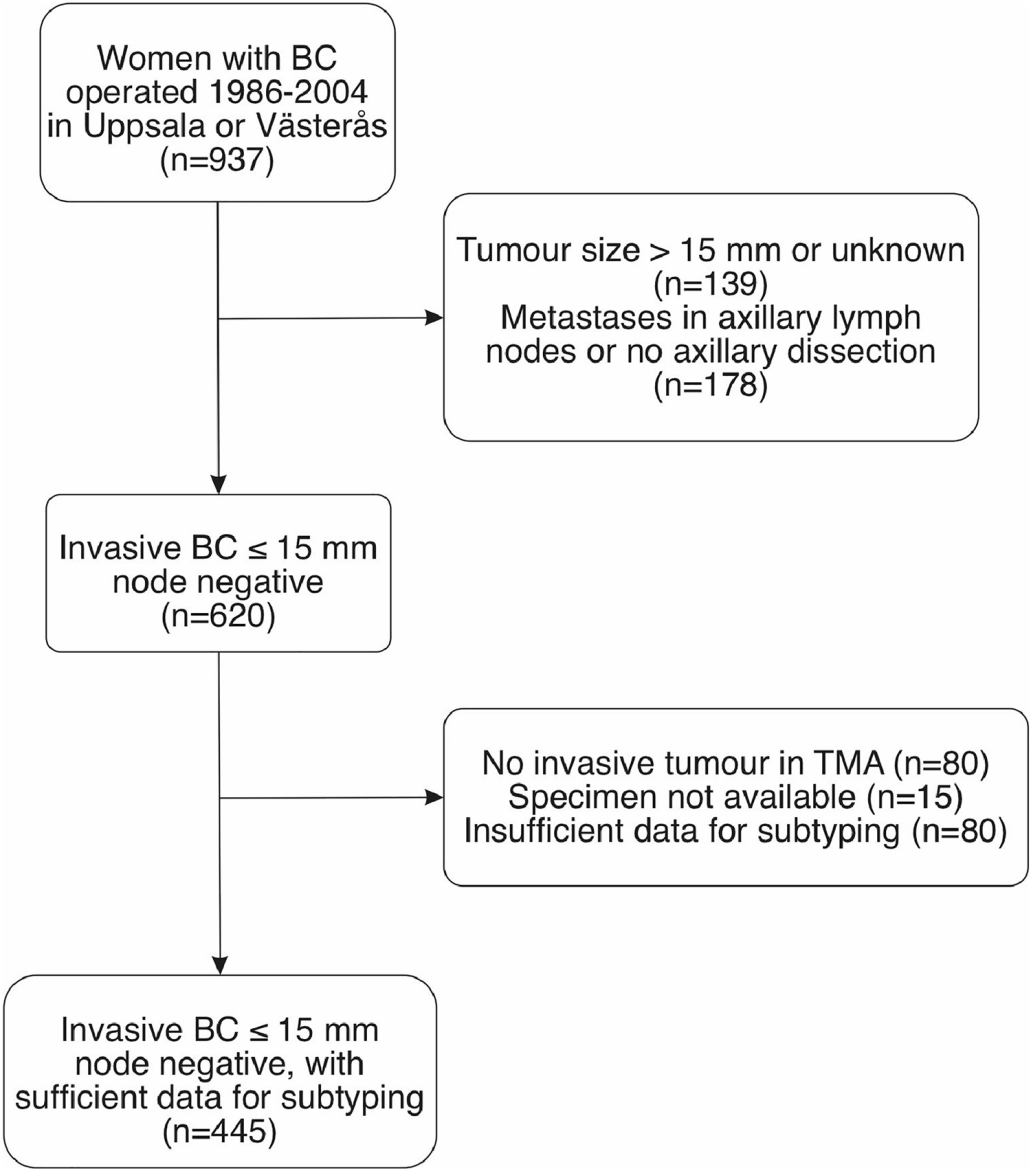
Selection of patients for the study cohort. *BC* breast cancer, *TMA* tissue microarray

A total of 937 women diagnosed with BC were identified through a search of the regional breast cancer quality of care registry together with the hospital records during the study period. Out of these, 620 women had an invasive tumour ≤ 15 mm without lymph node metastases on final histology, and 445 of these tumours had sufficient material in the TMA for subtyping (Fig. 1). Dropout analysis showed that tumours without sufficient material in the TMA were smaller (mean 9 mm vs. 10 mm, *p* < 0.001), slightly more often lobular (12% vs. 6.7%, *p* = 0.02) and patients received less endocrine therapy (13.7% vs. 22.5%), compared to the tumours with material available. There were no statistically significant differences in age, mode of detection, locoregional treatment or chemotherapy (Supplementary Table S1).

The follow-up time ranged from 0.2 to 29.6 years, with a median of 13.3 years. Median follow-up for women alive was 19.8 years. A high proportion of the tumours were Luminal A-like (59%) or Luminal B-like (HER2−) (28%). Within the group of luminal tumours with nuclear grade 2 (*n* = 267) 195 (73%) had low Ki-67, 36 (13.5%) had intermediate Ki-67 and 36 (13.5%) had high Ki-67. Most patients were diagnosed within the screening programme (71%), but women with Luminal B-like (HER2−), HER2-positive or TNBC more often had clinically detected tumours, compared to those with Luminal A-like tumours (34%, 42% and 37% vs. 25%, respectively) although this was not statistically significant (*p* = 0.08). Women with Luminal B-like (HER2−), HER2+, or TNBC tumours were younger than women with Luminal A-like tumours (mean age 59 years, 57 years, and 57 years vs. 62 years, respectively). Locoregional treatment did not differ between the groups. Most of the women (79%) received breast conserving surgery (BCS) in combination with radiotherapy (RT). A substantial minority (13%) were treated with BCS without RT, while the remainder received mastectomy with or without subsequent RT. Administration of systemic adjuvant therapy differed between groups, with endocrine therapy more often given to women with Luminal B-like (HER2−) BC, when compared to patients with Luminal A-like BC (27% vs. 23%). Very few patients (*n* = 9) received chemotherapy, most of those had HER2-positive BC (*n* = 3) or TNBC (*n* = 4) (Table 1).

**Table 1:** Cohort characteristics

	Whole cohort	Luminal A-like	Luminal B-like (HER2−)	HER2-positive	Triple negative		*p*-value
	*n* = 445	*n* = 264 (59%)	*n* = 125 (28%)	*n* = 26 (6%)	*n* = 30 (7%)		
Age years, mean (IQR)	61 (52–68)	62 (54–69)	59 (51–68)	57 (46–67)	57 (48–67)		0.009
< 50 years	85 (19%)	38 (14.4%)	28 (22.4%)	11 (42.3%)	8 (26.7%)		
≥ 50 years	360 (81%)	226 (85.6%)	97 (77.6%)	15 (57.7%)	22 (73.3%)		
Size, mm, mean, (IQR)	10 (8–13)	10 (8–12)	11 (8–14)	10 (7–11)	12 (10–15)		0.006
pT1a, *n* (%)	22 (5%)	15 (5.7%)	4 (3.2%)	3 (11.5%)	0		
pT1b	219 (49%)	139 (52.7%)	53 (42.4%)	14 (53.8%)	13 (43.3%)		
pT1c	204 (46%)	110 (41.7%)	68 (54.4%)	9 (34.6%)	17 (56.7%)		
Mode of detection							0.08
Screening	319 (71%)	199 (75.4%)	83 (66.4%)	15 (57.7%)	19 (63.3%)		
Clinical	129 (29%)	65 (24.6%)	42 (33.6%)	11 (42.3%)	11 (36.7%)		
Histological subtype^a^, *n* (%)							0.28
Ductal	393 (88%)	224 (84.8%)	118 (94.4%)	26 (100%)	25 (83.3%)		
Papillary/EPC	2 (0.4%)	1 (0.4%)	1 (0.8%)	0	0		
Lobular	30 (7%)	23 (8.7%)	4 (3.2%)	0	3 (10%)		
Mucinous	14 (3%)	12 (4.5%)	1 (0.8%)	0	1 (3.3%)		
Other	6 (1%)	4 (1.5%)	1 (0.8%)	0	1 (3.3%)		
Oestrogen receptors (ER)							< 0.001
ER+ (≥ 10%)	395 (88.8%)	254 (96.2%)	124 (99.2%)	17 (65.4%)	0		
ER− (< 10%)	37 (8.3%)	0	1 (0.8%)	8 (30.8%)	28 (93.3%)		
Missing	13 (2.9%)	10 (3.8%)	0	1 (3.8%)	2 (6.7%)		
Progesterone receptors (PR)							< 0.001
PR+ (≥ 10%)	326 (73.3%)	222 (84.1%)	93 (74.4%)	11 (42.3%)	0		
PR− (< 10%)	108 (24.3%)	36 (13.6%)	28 (22.4%)	14 (53.8%)	30 (100%)		
Missing	11 (2.5%)	6 (2.3%)	4 (3.2%)	1 (3.8%)	0		
Nuclear grade							< 0.001
1	41 (9.2%)	39 (14.8%)	0	2 (7.7%)	0		
2	292 (65.6%)	225 (85.2%)	42 (33.6%)	13 (50%)	12 (40%)		
3	112 (25.2%)	0	83 (66.4%)	11 (42.3%)	18 (60%)		
Locoregional treatment							0.56
BCS and RT	352 (79.1%)	202 (76.5%)	104 (83.2%)	21 (80.8%)	25 (83.3%)		
Mastectomy and RT	7 (1.6%)	5 (1.9%)	2 (1.6%)	0	0		
Mastectomy w/o RT	30 (6.7%)	16 (6.1%)	8 (6.4%)	3 (11.5%)	3 (10%)		
BCS w/o RT	56 (12.6%)	41 (15.5%)	11 (8.8%)	2 (7.7%)	2 (6.7%)		
Endocrine therapy							0.04
Yes	100 (22.5%)	60 (22.7%)	34 (27.2%)	5 (19.2%)	1 (3.3%)		
No	345 (77.5%)	204 (77.3%)	91 (72.8%)	21 (80.8%)	29 (96.7%)		
Chemotherapy							< 0.001
Yes	9 (2%)	0	2 (1.6%)	3 (11.5%)	4 (13.3%)		
No	436 (98%)	264 (100%)	123 (98.4%)	23 (88.5%)	26 (86.7%)		

*p*-values indicate level of significance for overall difference between subtype groups, using chi-square test for categorical variables and one-way ANOVA for continuous variables

*BC* breast cancer, *IQR* interquartile range, *EPC* encapsulated papillary carcinoma, *BCS* breast conserving surgery, *RT* radiotherapy

^a^Histologic subtype based on medical records

### Overall survival

**Fig. 2:**
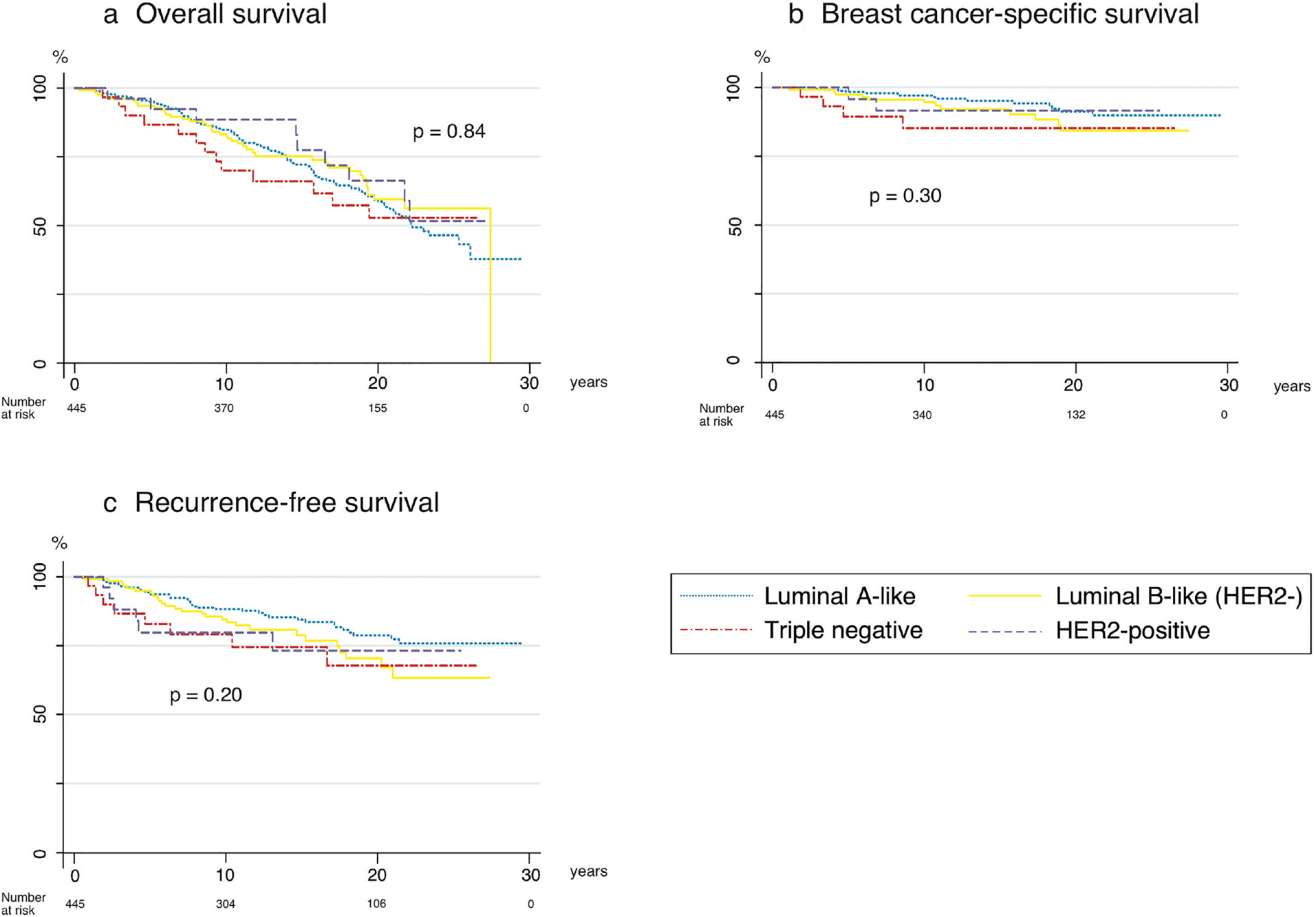
Survival outcomes (Kaplan Meier), **a** overall survival, **b** breast cancer-specific survival, **c** recurrence-free survival

A total of 168 women died, whereof 33 from breast cancer. The univariate analysis showed no statistically significant differences in OS between patients based on surrogate tumour subtypes (Fig. 2a). Multivariable analysis showed numerically increased hazard ratios (HR) for the non-Luminal A-like subtypes; 1.08 [Luminal B-like (HER2−); 95% confidence interval (CI) 0.75–1.55], 1.41 (TNBC; 95% CI 0.78–2.54), and 1.01 (HER2+; 95% CI 0.51–2.02) but the differences were not statistically significant. Clinical detection (HR 1.58; CI 1.12–2.23) and high age (HR 1.10; CI 1.08–1.12) increased the risk of dying. Table 2 summarizes the results of the multivariable analysis.

**Table 2:** Multivariable analysis of risk factors for overall survival

	*n*	HR (95% CI)	*p*-value
*Multivariable analysis of overall survival*			
Tumour subtype			
Luminal A-like	264	*Ref.*	
Luminal B-like (HER2−)	125	1.08 (0.75–1.55)	0.70
Triple negative	30	1.41 (0.78–2.54)	0.25
HER2-positive	26	1.01 (0.51–2.02)	0.97
Clinical detection^a^	129	1.58 (1.12–2.23)	0.01
Tumour size (mm)		1.01 (0.96–1.07)	0.71
Age (years)		1.10 (1.08–1.12)	< 0.001

*n* number of patients with risk factor, *HR* hazard ratio, *Ref.* reference, *RT* radiotherapy, *CI* confidence interval

^a^Compared to detection by screening

### Breast cancer-specific survival

Univariate analysis showed no statistically significant difference in BCSS between subtypes. (Fig. 2b). The 5-, 10-, and 20-year BCSS for the whole cohort were 98%, 96%, and 89%, respectively. Table 3 shows the survival rates for the respective subtypes. Of the 33 women who died from breast cancer, 15 had Luminal A-like tumours, 12 had Luminal B-like (HER2−) tumours, four had TNBC and two had HER2-positive tumours. In multivariable analysis, the non-Luminal A-like subtypes all had higher HRs than the Luminal A-like [Luminal B-like (HER2−) 1.53 (95% CI 0.70–3.33), TNBC 1.89 (95% CI 0.60–5.93), and HER2 + 1.23 (95% CI 0.28–5.40)] but the differences did not reach statistical significance (Table 4).

**Table 3:** Survival rates depending on molecular subtype

	*n*	5 years		10 years		20 years	
		OS (%)	BCSS (%)	OS (%)	BCSS (%)	OS (%)	BCSS (%)
*Cumulative survival rates depending on molecular subtype*							
All	445	94	98	84	96	60	89
LumA	209	95	98	85	97	60	91
LumB	180	94	97	83	96	59	83
TNBC	30	87	89	70	85	53	85
HER2+	26	96	100	88	91	68	91

*OS* overall survival, *BCSS* breast cancer specific survival, *LumA* Luminal A-like, *LumB* Luminal B-like (HER2−), *TNBC* triple negative breast cancer, *HER2+* HER2-positive

**Table 4:** Multivariable analysis of breast cancer-specific survival

	*n*	HR (95% CI)	*p*-value
*Multivariable analysis of breast cancer specific survival*			
Tumour subtype			
Luminal A-like	264	*Ref.*	
Luminal B-like (HER2−)	125	1.53 (0.70–3.33)	0.28
Triple negative	30	1.89 (0.60–5.93)	0.27
HER2-positive	26	1.23 (0.28–5.40)	0.79
Clinical detection^a^	129	1.46 (0.69–3.06)	0.32
Tumour size (mm)		1.08 (0.96–1.21)	0.21
Age (years)		0.99 (0.96–1.02)	0.47

*n* number of patients with risk factor, *HR* hazard ratio, *Ref*. reference, *CI* confidence interval

^a^Compared to detection by screening

### Recurrence-free survival

We found no statistically significant difference in RFS between subgroups in the present cohort either in univariate (Fig. 2c) or multivariable analysis (Table 5). The non-Luminal A-like subtypes, however all had higher estimated HRs compared to the Luminal A-like subtype [Luminal B-like (HER2−) 1.41 (95% CI 0.86–2.32), TNBC 1.66 (95% CI 0.76–3.63), and HER2 + 1.48 (95% CI 0.63–3.51)]. More extensive models for multivariable analysis of OS, BCSS, and RFS including treatment variables (radiotherapy, type of surgery, chemotherapy, and endocrine therapy) did not substantially change the estimated hazard ratios for the different subtypes (data not shown).

**Table 5:** Multivariable analysis of recurrence-free survival

	*n*	HR (95% CI)	*p*-value
*Multivariable analysis of recurrence-free survival*			
Tumour subtype			
Luminal A-like	264	*Ref.*	
Luminal B-like (HER2−)	125	1.41 (0.86–2.32)	0.17
Triple negative	30	1.66 (0.76–3.63)	0.21
HER2-positive	26	1.48 (0.63–3.51)	0.37
Clinical detection^a^	129	1.49 (0.92–2.40)	0.10
Tumour size (mm)		1.01 (0.94–1.09)	0.78
Age (years)		0.99 (0.97–1.01)	0.39

*n* number of patients with risk factor, *HR* hazard ratio, *Ref.* reference, *CI* confidence interval

^a^Compared to detection by screening

### Locoregional and distant recurrence

**Fig. 3:**
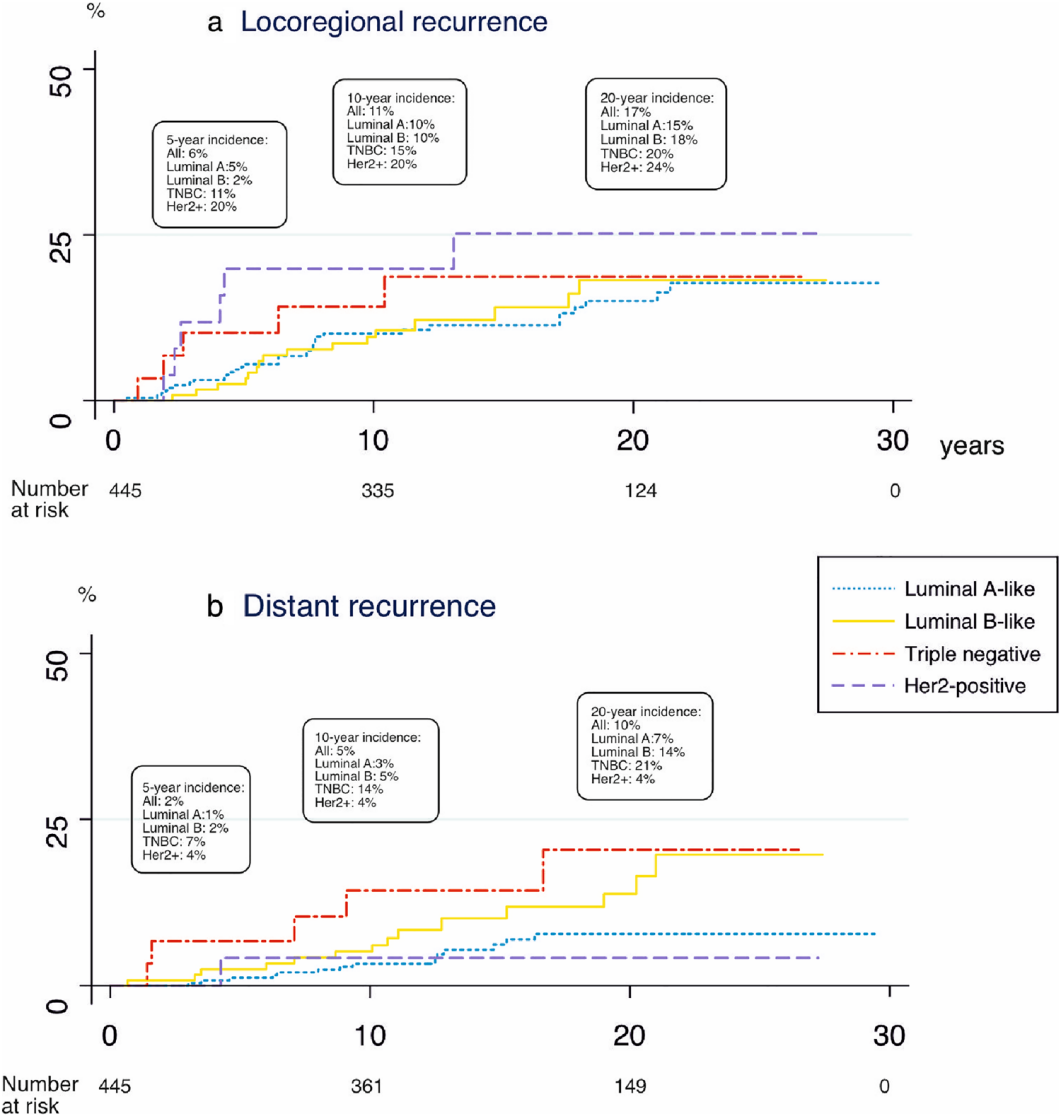
Cumulative incidence of locoregional (**a**) and distant recurrence (**b**)

In total 60 women (13%) had locoregional recurrence and 34 women (8%) had distant metastasis. We found no statistically significant difference in locoregional recurrence across subtypes (Fig. 3a). By contrast, there was a difference in the incidence of distant metastasis (*p* = 0.03) where the TNBC (HR 3.24; 95% CI 1.17–9.00) and Luminal B-like (HER2−) (HR 2.26; CI 1.08–4.75) had more distant metastases as compared with the Luminal A-like subtype. Distant recurrence occurred early in TNBC, and later in the Luminal B-like (HER2−) BC, hence the difference between these two groups gradually diminished with longer follow-up (Fig. 3b).

## Discussion

No certain association of surrogate molecular subtypes with overall survival was found in this population-based cohort of women with small node negative breast cancers (BC) and a very long follow-up time. Women with small TNBC or Luminal B-like (HER2−) tumours were however three times (TNBC) or twice [Luminal B-like (HER2−)] as likely to have had a distant recurrence compared to woman with a Luminal A-like tumour after 20 years. These women also had worse BCSS compared to women with Luminal A-like tumours, although the difference was not statistically significant. Women with HER2+ tumours had neither worse BCSS, nor higher incidence of distant recurrence than the women with Luminal A-like tumours, despite not receiving any targeted anti-HER2 therapy.

### TNBCs are more aggressive and may merit systemic therapy even when they are small

Women with TNBC fared worse than women with Luminal A-like tumours in terms of OS, BCSS and RFS although differences were statistically significant only for distant recurrences. Our findings are supported by a SEER database study showing that women with pT1abN0 ER− tumours more often died from BC and those with ER+ more often of other causes [15], as well as other studies showing that TNBC is associated with worse RFS and distant RFS in pT1abN0 [16] or pT1bN0 tumours [9]. Thus, it would seem prudent to consider chemotherapy even for small node negative TNBC. Indeed, results from older prospective trials show a benefit of chemotherapy for ER− pT1abN0 tumours [17]. Several newer retrospective studies, however, failed to show any association between chemotherapy and outcome in pT1abN0 TNBC tumours [16, 18, 19], emphasizing the need for prospective trials on this group of patients, receiving modern locoregional therapy and using relevant definitions of hormone receptor status and HER2-status.

### Small HER2-positive tumours have a favourable prognosis even in the absence of HER2-targeted therapy

The most surprising finding in our study was that HER2-positive tumours did not have a significantly worse long-term outcome compared to Luminal A-like tumours, even though no patient received targeted HER2 therapy. The numbers are small and warrant a cautious interpretation but are nonetheless interesting. HER2-positivity had no association with OS, possibly because women with HER2+ tumours were younger on average, a finding consistent with other studies [10, 20]. The BCSS was worse compared to Luminal A-like tumours after 10 years (91% vs. 97%) but after this the difference evened out. The only analysis where the HER2+ tumours stood out was in the locoregional recurrences where the cumulative incidence was fourfold higher after five years and still almost twice as high compared to Luminal A-like tumours after 20 years. Other retrospective studies have also failed to show HER2-positivity in pT1abN0 BC being an independent factor for worse DFS [20] or distant RFS [8]. In contrast, two retrospective studies have shown worse RFS and distant RFS for HER2 + pT1abN0 tumours [7] or pT1bN0 tumours [9], respectively. The second study did not, however, find any worse outcome for HER2 + pT1aN0 tumours. Another retrospective study described worse RFS and BCSS for HER2 + pT1mic/ab N0 tumours [6].

One of the reasons for the conflicting results in the literature may be how the molecular surrogate HER2-positive group is defined. By the original definition, “HER2-enriched” is ER-negative [2] but many authors define it as HER2 + regardless of hormone receptor status, as was also the case for the present cohort. The rationale behind this is that treatment recommendations generally do not consider ER-status in HER2 + BC [21, 22]. In our cohort, most (17/26) HER2 + tumours were ER+, but the BC deaths and the distant recurrences all occurred in the HER2+/ER− subgroup, while locoregional recurrences occurred equally in the HER2+/ER− and HER2+/ER+ groups (data not shown). Furthermore, there are retrospective studies showing a benefit of endocrine therapy alone (for HER2+/ER+ tumours) but not chemotherapy with/without trastuzumab, for women with HER2-positive pT1abN0 tumours [19, 20]. A retrospective study describing a better OS for patients with HER2-positive pT1abN0 tumours receiving trastuzumab and chemotherapy [23] included women who received only chemotherapy, only endocrine therapy or no adjuvant treatment at all in the same comparison group making the results somewhat hard to disentangle. Together with our findings, this suggests that small node negative HER2+ tumours have a good prognosis if they are ER+, and hence that the benefits of adding chemotherapy and HER2-targeted therapy on top of endocrine therapy may not be substantial.

### Outcome of luminal B-like (HER2−) tumours depends strongly on the length of follow-up

The natural course of BC varies depending on subtype, where the Luminal-like tumours have a slow but steady rate of recurrence and death over the years, while the HER2+ and TNBC have a higher mortality rate initially, which declines after the first 5 years [24, 25]. The rate of recurrence and death for Luminal B-like (HER2−) tumours is only slightly higher than for the Luminal A-like, with almost no difference at five years of follow-up, but after 20 years actually being on the same level as TNBC. In our cohort the 5-year BCSS were similar 98% (Luminal A-like) and 97% [Luminal B-like (HER2−)], but after 20 years had diverged to 91% (Luminal A-like) and 83% [Luminal B-like (HER2−)]. The same held true for both locoregional and distant recurrences. Our findings are consistent with a recent registry-based study with long-term follow-up in which Luminal B-like (HER2−) and TNBC eventually had the same risk of BC events [26]. Only a third of the women with Luminal B-like (HER2−) tumours in the present cohort received endocrine therapy and a tenth received lumpectomy without subsequent RT, which certainly influenced the long-term outcome [27]. Today, endocrine therapy would have been recommended for all of them, and likely none would have had BCS without RT. These results suggest that the possible benefit from addition of chemotherapy is highly dependent on the expected life span of the woman.

### Limitations

The strength in having a cohort with very long follow-up confers a limitation in that old specimens are used. It is known that the levels of detectable protein in archival FFPE tissue decrease over time but also that many proteins are still detectable after 70 years [28–31]. For each biomarker the effect of analysing TMA rather than whole slides also has to be considered, although since the tumours in our cohort are small, this risk is probably lower than average. For ER the effect of aging should be negligible due to its bimodal distribution [33, 34] and even with a 10% decrease per decade [32] the vast majority of ER+ tumours would still be scored as ER+. For the same reason, concordance between TMA and whole slide evaluation is assumably excellent [35, 36]. This is confirmed by comparison with the available original pathology reports, showing that 95% of the ER+ tumours were classified as ER+ by TMA-scoring (data not shown). For PR the distribution is somewhat different [37], leading to discrepancy between cores in 7% of cases in one study [35], possibly leading to misclassification of a small number of Luminal tumours with intermediate proliferation.

For Ki67, the decrease is perhaps more problematic and could also affect the subdivision between Luminal A-like versus Luminal B-like (HER2−). In our material there were indeed slightly more Luminal B-like (HER2−) tumours in the samples from more recent years, (data not shown) The proportion of HER2-positive tumours in our cohort was as expected in a sample of small tumours [6, 20] even though this is the most intratumourally heterogenous stain [28, 35]. Since we performed SISH on all samples and DNA is thought to be more stable than membrane proteins [32], as well as less heterogenous [38, 39] we consider the risk that we missed any HER2-positive cases very low. Still, the small number of HER2-positive tumours prohibited further subdivision of these patients into ER+ and ER− groups. Likewise, the number of TNBC were low, as was the number of events in each group. While this underscores the generally good prognosis for these women, it makes it difficult to reach statistical significance. Using surrogate molecular subtypes, rather than the actual molecular subtypes based on gene expression analysis may also be considered a weakness, since these do not correlate perfectly [40]. On the other hand, surrogate molecular subtypes are currently used in clinical practice in many settings and the molecular subtypes can also vary depending on signature and gene expression test used [41]. In summary, our limitations all mainly affect the subdivision between Luminal A-like and Luminal B-like (HER2−) breast cancer. However, the resulting groups still had measurably different outcomes. The clinicopathological factors used to guide treatment during the recruitment period of the cohort correlates with breast cancer subtypes and may have influenced analysis of the association between prognosis and subtype. At the time of treatment, modern protocols were however not in practice and few women were given adjuvant systemic treatment.

### Strenghts

The strengths of this study include a very long follow-up with no patients lost to follow-up. This is in contrast with most of the currently available studies for this patient group, and essential for analysing the outcome of the Luminal B-tumours. This means that the accuracy of the findings is high, compared to the data that may be extracted from a registry. The cohort is population based, and the analyses of TMA data include more than 70% of the eligible women, making it representative for women with small lymph node negative BC at that time. There was no difference in primary surgery or RT between the groups, indicating that they were probably comparable with respect to co-morbidity. None of the women with HER2-positive tumours were treated with targeted therapy, making it possible to observe the natural course of these tumours. Finally, the histological evaluation was done by the same two subspecialised breast pathologists, reducing interobserver variation.

## Conclusion

Small TNBC and Luminal B-like (HER2−) tumours behave more aggressively than Luminal A-like tumours. These subtypes follow different courses, where the TNBC recur mostly early on or not at all, while the Luminal B-like (HER2−) tumours recur at a slow but consistent rate over the years. This means that a young and otherwise healthy woman with a stage 1 Luminal B tumour might benefit substantially from systemic adjuvant therapy, while for an older woman the risks may outweigh the benefits. For early stage TNBC, our study confirms a high 10-year risk of recurrence and death, and thus patients should stand to gain from systemic adjuvant therapy. For the women with HER2+ tumours however, neither our findings, nor the available literature unequivocally support an increased risk of distant metastasis or death for pT1abN0-tumours in absence of HER2-targeted treatment. It is possible that treatment recommendations for HER2+ tumours need to take ER-status into account, and that women with ER+/HER2+ tumours have no need for adjuvant therapy in addition to locoregional radiotherapy and endocrine treatment. Despite the different risks associated with the subtypes described above, no association of tumour subtype with OS was observed. Prospective trials using modern locoregional therapy and endocrine therapy are thus needed to evaluate whether the more aggressive behaviour of non-Luminal A-like subtypes translate into a benefit of systemic adjuvant chemotherapy and HER2-targeted therapy for women with small lymph node negative BC.

## Supplementary Information

Below is the link to the electronic supplementary material.

Supplementary file 1 (DOCX 16 kb)

## Data Availability

Data is not uploaded to a publicly available platform. Researchers have access to data through application to the study PI (malin.sund@umu.se) or the corresponding author under standard rules of protecting data integrity and existing ethics permissions.
