# Supplementary material for: Correlation of tumour subtype with long-term outcome in small breast carcinomas: a Swedish population-based retrospective cohort study
Source: Breast Cancer Res Treat. 2022 Aug 6;195(3):367–77. doi: 10.1007/s10549-022-06691-4 (PMC9464733; doi:10.1007/s10549-022-06691-4)
Supplement: Supplementary file 1 — (DOCX 16 kb) [file 10549_2022_6691_MOESM1_ESM.docx]

***Supplementary table 1 Dropout analysis***

|  | Patients represented in TMA  n=445 | Patients not included in TMA-analysis*  n=175 |  | *p-value* |
| --- | --- | --- | --- | --- |
| Age yrs, mean (IQR)   - < 50 yrs - ≥ 50 yrs | 61 (52-68)  85 (19%)  360 (81%) | 59 (50-67) |  | 0.09 |
| Size, mm, mean, (IQR)   - pT1a, n (%) - pT1b - pT1c | 10 (8-13)  22 (5%)  219 (49%)  204 (46%) | 9 (6-12)  33 (18.9%)  90 (51.4%)  52 (29.7%) |  | <0.001 |
| Mode of detection   - Screening - Clinical | 316 (71%)  129 (29%) | 124 (71%)  51 (29%) |  | 0.27 |
| Histological subtype,° n (%)   - Ductal - Papillary/ EPC - Lobular - Mucinous - Other - Missing | 393 (88.3%)  2 (0.4%)  30 (6.7%)  14 (3.1%)  6 (1.3%)  0 | 142 (81.1%)  0  21 (12.0%)  6 (3.4%)  1 (0.5%)  5 (2.9%) |  | 0.02 |
| Locoregional treatment   - BCS and RT - Mastectomy and RT - Mastectomy w/o RT - BCS w/o RT | 352 (79.1%)  7 (1.6%)  30 (6.7%)  56 (12.6%) | 129 (73.3%)  6 (3.4%)  20 (11.4%)  20 (11.4%) |  | 0.1 |
| Endocrine therapy   - Yes - No | 100 (22.5%)  345 (77.5%) | 24 (13.7%)  151 (86.2%) |  | 0.02 |
| Chemotherapy   - Yes - No | 9 (2%)  436 (98%) | 2 (1.1%)  173 (98.9%) |  | 0.46 |

**Node negative patients with tumours ≤ 15 mm, excluded from TMA-analysis because specimen was unavailable, there was no invasive tumour in TMA or because material was not sufficient for complete subtyping.*

*°Histologic subtype based on medical records.*
